# Evaluating the Potential of Younger Cases and Older Controls Cohorts to Improve Discovery Power in Genome-Wide Association Studies of Late-Onset Diseases

**DOI:** 10.3390/jpm9030038

**Published:** 2019-07-22

**Authors:** Roman Teo Oliynyk

**Affiliations:** 1 Centre for Computational Evolution, University of Auckland, Auckland 1010, New Zealand; roli573@aucklanduni.ac.nz; 2 Department of Computer Science, University of Auckland, Auckland 1010, New Zealand

**Keywords:** GWAS, genome-wide association studies, genetics, polygenic risk score, heritability, late-onset disease, simulation, gene variant, SNP

## Abstract

For more than a decade, genome-wide association studies have been making steady progress in discovering the causal gene variants that contribute to late-onset human diseases. Polygenic late-onset diseases in an aging population display a risk allele frequency decrease at older ages, caused by individuals with higher polygenic risk scores becoming ill proportionately earlier and bringing about a change in the distribution of risk alleles between new cases and the as-yet-unaffected population. This phenomenon is most prominent for diseases characterized by high cumulative incidence and high heritability, examples of which include Alzheimer’s disease, coronary artery disease, cerebral stroke, and type 2 diabetes, while for late-onset diseases with relatively lower prevalence and heritability, exemplified by cancers, the effect is significantly lower. In this research, computer simulations have demonstrated that genome-wide association studies of late-onset polygenic diseases showing high cumulative incidence together with high initial heritability will benefit from using the youngest possible age-matched cohorts. Moreover, rather than using age-matched cohorts, study cohorts combining the youngest possible cases with the oldest possible controls may significantly improve the discovery power of genome-wide association studies.

## 1. Introduction

With a growing fraction of the population reaching advanced age, late-onset diseases (LODs) have become the leading cause of mortality and morbidity [1]. Some LODs such as macular degeneration [2,3,4] are primarily caused by a single or a small number of high-effect variants. Each such disease is individually relatively rare in the population, and the mutations causing the majority of such diagnoses are known [5]. The OMIM Gene Map Statistics [6] compendium lists thousands of such gene mutations.

The most common LODs are polygenic. They include heart disease, cancer, respiratory disease, stroke, and notably, Alzheimer’s disease and other dementias [7]. The object of genome-wide association studies (GWASs) is to detect associations between genetic variants and traits in population cohorts [8]. Associations can be used to predict individuals’ LOD liability and, based on this knowledge, formulate preventive recommendations and treatments, with the ultimate goal of applying personalized medical interventions based on the genetic makeup of each unique individual [8]. GWASs are being applied to all areas of genetics and medicine. Yet polygenic LODs remain resistant to the discovery of sufficient causal gene variants that would allow for accurate predictions of an individual’s disease risk [3,9,10]. GWASs can implicate only a subset of single nucleotide polymorphisms (SNPs) that can typically explain a fraction of the heritability of a polygenic LOD [8], despite the fact that LODs with varied symptoms and phenotypes show high heritability in twin and familial studies [11,12,13,14,15,16,17,18,19].

Two complementary scenarios can explain LOD heritability, and both contribute to the so-called GWASs’ missing heritability problem [20,21,22,23]. The common low-effect-size allele hypothesis states that LODs are primarily caused by a combination of a large number of relatively common alleles of small effect [24]. GWASs have been able to discover only a small number of moderate-effect SNPs, but a large number of smaller effect SNPs remain below GWASs’ statistical discovery power. The rare high-effect-size allele hypothesis proposes that LODs are caused by a relatively small number of rare, moderate- or high-effect alleles with a frequency below 1% that likely segregate in various proportions into subpopulations or families [25,26] and are similarly problematic for GWASs’ discovery. Both scenarios can contribute to observational facts, but their relative weights vary depending on the genetic architecture of an LOD [27]. It has been determined [28,29] that common low-effect-size variants very likely explain the majority of heritability for most complex traits and LODs. This study primarily focuses on such diseases.

Recently, Warner and Valdes [30] stated that “one of the criticisms raised against genetic studies is that they are far removed from clinical practice”. Performing GWASs with ever-larger cohort sizes achieves better and more complete discovery for a variety of LODs and traits, yet larger patient cohorts are associated with practical, logistic, ethical, and financial limitations, and research continues on developing statistical and procedural methods to improve discovery efficiency and sensitivity. Traditionally, homogeneity of cohort participants is recommended for GWAS. A common approach is to adjust for known covariates, including age, with the goal of correcting or averaging out biases [31]. Several studies caution about the appropriateness and scope of covariate adjustments [32,33]. Usually, the same age window is targeted, although it has been suggested [34] that individuals with an early age of onset are likely to have greater genetic susceptibility. Li and Meyre [34] proposed that once the risk of false positive association has been ruled out by initial replication studies, association can be extended to different age-matched windows. The recognition that “extreme phenotype sampling” may improve GWAS discovery prompted theoretical interest in study cohorts that are diverse in age [35,36].

A recent study [37] simulated population age progression under the assumption of relative disease liability remaining proportionate to individual polygenic risk and determined that individuals with higher risk scores will become ill and be diagnosed proportionately earlier, bringing about a change in the distribution of risk alleles between new cases and the as-yet-unaffected population in every subsequent year of age. This is accompanied by a lowering of the mean polygenic risk score (PRS) of the progressively older as-yet-unaffected population and impairment of GWASs’ statistical discovery power for the study cohorts comprised of older age-matched individuals, most prominently for the highest prevalence LODs.

The simulations were based on Cox’s proportional hazards model [38], where the probability of developing a disease at a particular age, given that a subject has been disease-free until that age is given by the multiplicative effect of a set of risk factors over the baseline hazard of the disease [39] (see also the discussion accompanying Equation (1) in the Methods). According to Chatterjee et al. [39], “to date, post-GWAS epidemiological studies of gene-environment interactions have generally reported multiplicative joint associations between low-penetrant SNPs and environmental risk factors, with only a few exceptions”, and “investigations of SNP-by-SNP and SNP-by-environment interactions using data from large GWAS generally suggest that the assumption of multiplicative effects is often adequate and an additive model under the identity link can be soundly rejected”; studies [40,41,42,43] provide corroboration of these conclusions.

This research quantifies the use of non-age-matched cohorts for improving the discovery power of GWASs using as a case study eight prevalent LODs: Alzheimer’s disease (AD), type 2 diabetes (T2D), coronary artery disease (CAD), cerebral stroke, and four late-onset cancers: breast, prostate, colorectal, and lung cancer. The simulation results showed that GWASs of polygenic LODs that display both high cumulative incidence at older age and high initial familial heritability may benefit most from using the youngest possible participants as cases. Additional improvement in GWASs’ discovery power could be achieved by study cohorts that combine the youngest possible cases with the oldest possible controls.

## 2. Materials and Methods

### 2.1. The Simulation Design Summary and Conceptual Foundations

This study’s simulations are an extension of the author’s earlier research [37] that focused on the allele frequency and GWASs’ statistical power change patterns in aging populations for the eight LODs that were further analyzed here: Alzheimer’s disease, type 2 diabetes, coronary artery disease, cerebral stroke, and four late-onset cancers—breast, prostate, colorectal, and lung cancer. A brief summary that includes excerpts from the Methods section of the earlier publication describing the model genetic architectures, the LOD incidence models, the statistical foundations, and the simulation overview are provided in this subsection. Please see the Methods section in [37] for a more complete treatment. Section 2.2 and Section 2.3 will describe the simulation design and analysis that was performed exclusively in this study.

According to Chatterjee et al. [39], the conditional age-specific incidence rate of the disease, I(t|G), which is defined as the probability of developing the disease at a particular age *t*, given that a subject has been disease-free until that age, can be modeled using Cox’s proportional hazards model [38]:

(1) $$I\left(t|G\right)=I_{0}\left(t\right)exp\left(\sum_{k}b_{k}G_{k}\right),$$

where $G=(G_{1},\cdots ,G_{k})$ is the multiplicative effect of a set of risk factors on the baseline hazard of the disease $I_{0}\left(t\right)$. The set of age-independent variables in *G* could include genetic and environmental risk factors, as well as their interaction terms.

The following summary from Chatterjee et al. [39] is particularly relevant to the methodology of this research: “logistic regression methods are preferred for the evaluation of multiplicative interactions. For case–control studies, if it can be assumed that environmental risk factors are independent of the SNPs in the underlying population, then case-only and related methods can be used to increase the power of tests for gene–environment interactions. To date, post-GWAS epidemiological studies of gene–environment interactions have generally reported multiplicative joint associations between low-penetrant SNPs and environmental risk factors, with only a few exceptions”. This means that the polygenic score $G=\sum_{k}b_{k}G_{k}$, as the lifelong characteristic of each individual, is used multiplicatively with $I_{0}\left(t\right)$, which encompasses environmental and aging effects. The simulations in this study used the functional approximations of the yearly incidence of Alzheimer’s disease, type 2 diabetes, coronary artery disease, cerebral stroke, and four late-onset cancers: breast, prostate, colorectal, and lung cancer.

Five genetic architecture scenarios were analyzed in [37], and by comparing the patterns characteristic to each of these architectures, as well as extensive validation simulations, it was determined that the common low-effect genetic architecture, as indeed is the current scientific consensus [28,29], best fits the clinical and familial studies observations, and the analysis here is based exclusively on this architecture (although not discussed here, Supplementary Data also contains simulations and analysis results for rare medium-effect-size allele architecture).

In the case of the common low-effect genetic architecture, the minor allele frequencies (MAFs) are distributed in equal proportions at 0.073, 0.180, 0.286, 0.393, and 0.500, while the odds ratio (OR) values are 1.15, 1.125, 1.100, 1.075, and 1.05, resulting in 25 combinations. Having multiple well-defined alleles with the same parameters facilitated the tracking of their behaviors with age, LOD, and simulation incidence progression.

An individual polygenic risk score β can be calculated as the sum of the effect sizes of all alleles, which is by definition a log(OR) (natural logarithm of odds ratio) for each allele, also following Pawitan et al. [44]:

(2) $$\beta =log\left(OR\right)=\sum_{k}a_{k}log\left(OR_{k}\right),$$

where $a_{k}$ is the number of risk alleles (0, 1 or 2) and $OR_{k}$ is the odds ratio of additional liability presented by the *k*-th allele. Variance of the allele distribution is determined by

(3) $$var=2\sum_{k}p_{k}\left(1-p_{k}\right){\left(log\left(OR_{k}\right)\right)}^{2},$$

where $p_{k}$ is the frequency of the *k*-th genotype [44]. The contribution of genetic variance to the risk of the disease is heritability:

(4) $$h^{2}=\frac{var}{var+\pi^{2}/3},$$

where $\pi^{2}/3$ is the variance of the standard logistic distribution [45]. For example, the number of variants needed for the Scenario A LODs is summarized in Table 1.

Following Pawitan et al. [44], the variants are assigned to individuals with frequencies proportionate to MAF $p_{k}$ for SNP *k*, producing, in accordance with the Hardy–Weinberg principle, three genotypes (AA, AB, or BB) for each SNP with frequencies $p_{k}^{2}$, $2p_{k}\left(1-p_{k}\right)$, and ${\left(1-p_{k}\right)}^{2}$. The mean value $\beta_{mean}$ of the population distribution can be calculated using the following equation:

(5) $$\beta_{mean}=2\sum_{k}p_{k}log\left(OR_{k}\right).$$

In this prospective simulation, each next individual to be diagnosed with an LOD is chosen proportionately to that individual’s relative PRS at birth, relative to all other individuals in the as-yet-unaffected population. The number of individuals diagnosed annually is determined using the model incidence rate curve derived from clinical statistics. In this manner, the aging process is probabilistically reproduced using a population simulation model rather than a computational model. As the simulation progresses, the risk alleles are tracked for all newly diagnosed individuals and the remaining unaffected population, and their representation in the affected and remaining population is statistically analyzed. For each such allele in the simulated population, the allele frequency for cases and controls is tracked as age progresses. The non-centrality parameter (NCP) λ can be calculated following Vukcevic et al. [46]:

(6) $$\lambda =2N\theta \left(1-\theta \right)f\left(1-f\right)\beta^{2},$$

where *N* is the overall population sample size, θ is the fractions of cases, and (1-θ) is the fraction of controls. The value θ=0.5, or an equal number of cases and controls, is used throughout this publication. The sampled population MAF *f* and the effect size β=log(OR) for an allele of interest are determined from the cases and controls allele frequencies.

Having obtained the NCP λ from Equation (6), Luan et al. [47] recommended using SAS or similar statistical software to calculate the statistical power, using the following SAS statement (an equivalent R statement was implemented in this study):

(7) StatPower=1-PROBF(FINV(PSign,1,N-4),1,N-4,λ),

where PSign=0.99999995 corresponds to the $5\times 10^{-8}$ genome-wide significance level common in GWASs.

The comprehensive description of all simulation procedures and validation scenarios is available in [37].

### 2.2. Simulations and Analysis of the Youngest Possible Cases and Older Controls Cohorts Scenario

For the purposes of this research, rather than analyzing only the age-matched cohorts, the simulation progressed in age until the mid-cohort age at which the fraction of population that succumbed to an LOD exceeded 0.25% population prevalence—the prevalence that was postulated as a minimum needed for forming the cases cohorts. This set of diagnosed individuals was kept for the duration of the simulation as the cases cohort. The cohort’s age span was fixed at 10 years just as in the preceding study, a relatively common cohort age span in GWASs. The simulation continued with population aging and being subject to probabilistic disease incidence, and at each progressive year of age, a new random set of as-yet-unaffected individuals was sampled, thereby forming a new cohort with a progressively higher mid-cohort age. These cases and controls cohorts were analyzed for the effect allele frequency difference between cases and controls, with the corresponding estimate of the cohort size needed to achieve 80% GWASs statistical discovery power. After completion of each simulation run with a mid-cohort age exceeding 100 years, the results were aggregated and further analyzed.

### 2.3. GWASs Association Analysis and Effect-Size Adjustment for Younger Cases and Older Controls Cohorts

The case–control populations produced by these simulations were suitable for the consequential GWASs association analysis that was implemented in this research. The simulations described in Section 2.1 were extended to save the output in PLINK format [48,49]. The initial validation, analysis, and file format conversions were performed using PLINK v1.9. The GWASs logistic regression with adjustment for age was performed using the R script *AdjustByAge.R*, as described below, and the outputs were validated with the regression modeling strategies (rms) GWASs R package by Harrell Jr. [50] and PLINK, confirming that the individual SNP association results with these two programs were identical to those produced by this R script.

The GWASs simulations showed that the apparent effect size tended to increase with the age of the control cohort, when analyzed against the youngest possible case cohort, compared to a “true” value, which was chosen as the effect size value from the youngest age-matched cohort. An example, although with a different objective, was demonstrated by the application of age bias in a leprosy case–control study [51] that used the bias adjustment as a function of squared age.

The R script written for this analysis, *AdjustByAge.R*, based on R generalized linear model glm() functionality [52], performed the GWASs association and iterative age covariate adjustment starting with a youngest possible age-matched cohort and proceeding with the progressively older control cohorts. The script effectively discovered the best match bias adjustment power and allowed the comparison of the power parameters analyzed between LODs, as presented in the Results section. Importantly, the bias adjustment results showed that the increase in the value of the effect size was approximately proportionate to the effect size magnitude for all LODs analyzed here. The differential normalized effect size D(t) can be expressed as:

(8) $$D\left(t\right)=\left(\beta \left(t\right)-\beta_{True}\right)/\beta_{True}=\Delta \beta \left(t\right)/\beta_{True},$$

where β(t) and $\beta_{True}$ are the effect-size values found for older control cohorts compared to a known “true” effect size as defined in the simulated genetic architecture for each allele. The variable D(t) will be referred to as normalized bias. The GWASs simulations associated the effect sizes in 5-year control cohort age increments and matched the best power exponent regression function:

(9) $$D\left(t\right)=I_{0}+S{\left(t-Age_{Y}\right)}^{P},$$

where $I_{0}$ and *S* are the linear regression intercept and slope, *t* is an older control cohort age, $Age_{Y}$ is the youngest case cohort age, and *P* is the best match power exponent. When the solution to Equation (9) is correctly estimated for one gene variant (likely for a SNP with a larger effect size), it could be used to adjust other discovered variants’ effect sizes from Equations (8) and (9). The R script *FindAdjustmentRegressionFunction.R*, implementing lm() linear regression iteratively, fitted the best matching adjustment power with lowest residuals. Additionally, this script evaluated the regression with fixed *P* = 2 (quadratic regression) for all LODs. The data preparation and scripting steps described here are listed with specifics in *GwasSimulationPipeline.txt*, available along with the R scripts in the Supplementary Data.

### 2.4. Data Sources, Programming, And Equipment

The population mortality statistics from the US Social Security Actuarial Life Table [53] provided yearly death probability and survivor numbers up to 119 years of age for both men and women. Disease incidence data from the following sources were extensively used for analysis, using the materials referenced in supplementary Chapter S1 in [37]: Alzheimer’s disease: [12,54,55,56]; type 2 diabetes: [57]; coronary artery disease and cerebral stroke: [58]; and cancers: [59,60].

The simulations were performed on an Intel Xeon Gold 6154 CPU-based 36-core computer system with 288 GB of RAM. The simulation is written in C++ and can be found in the Supplementary Data. The simulations used population pools of 2 billion individuals for the LOD simulations and 300 million for validation simulations, resulting in minimal variability in the results between runs. The cohort simulations were built sampling at minimum 5 million cases and 5 million controls from the surviving portion of the initial 2 billion simulated individuals, which is equivalent to 0.25% of the initial population. This means that the cohort study began its analysis only when this cumulative incidence was reached. Conversely, the analysis ceased when, due to mortality, the number of available cases or controls declined below this threshold. For all LODs, this maximum mid-cohort age was at least 100 years and, depending on LOD, up to a few years higher. This confirms that, as described in the Discussion section, in cohorts composed of younger cases and older controls, it is feasible to form control cohorts of up to 100 years of age.

The simulation runs for either all validation scenarios or for a single scenario for all eight LODs took between 12 and 24 h to complete. The results represent the single final run of the simulation. To calculate the variability between runs, the simulations were re-run 16 times, and the two-sigma confidence interval for variability between runs is summarized in the statistical analysis below. The final simulation data, additional plots and elucidation, source code, and the Windows executable are included in the Supporting Information. Intel Parallel Studio XE was used for multi-threading support and Boost C++ library for faster statistical functions; the executable may be built and can function without these two libraries, with a corresponding slowdown in execution. The ongoing simulation results were saved in comma separated files and further processed with R scripts during subsequent analysis, also available in the Supplementary Data.

### 2.5. Statistical Analysis

Large variations between simulation runs complicate the analysis of population and genome models. This issue was addressed in this study by using a large test population, resulting in negligible variability between runs. The statistical power estimates deviated less than 1% in a two-sigma (95%) confidence interval, except for the early Alzheimer’s disease cohort, which commenced at 1.5% and fell below the 1% threshold within 4 years (see *TwoSDFraction.csv files in the Supplementary Data). In addition to ensuring that the simulations operated with reliable data, this eliminated the need for the confidence intervals in the graphical display.

The GWASs simulations and variant effect size covariate adjustment by age were more memory-intensive, and the 200 million simulated population with 500 thousand case and control cohorts was possible with the described equipment. In this instance, two-sigma confidence intervals for simulated GWASs discovery and regression parameters are presented in the corresponding plots.

## 3. Results

### 3.1. Impairment of GWASs’ Statistical Discovery Power with Progressively Older Age-Matched Cohorts

The preceding study [37] reported the patterns of GWASs’ discovery power for the age-matched cohorts. Out of the range of genetic architectures, simulation scenarios, and validations performed in that study, it is necessary to refer to the findings for the common low-effect-size genetic architecture, which are summarized in this subsection for further comparisons.

In this prospective simulation, each next individual to be diagnosed with an LOD was chosen proportionately to that individual’s relative PRS at birth relative to all other individuals in the as-yet-unaffected population, with the number of individuals diagnosed annually determined by the model incidence rate curve derived from clinical statistics. The simulation continued with population aging and being subject to probabilistic disease incidence, and at each progressive year of age, a new random set of as-yet-unaffected individuals was sampled, thereby forming a new cohort with a progressively higher mid-cohort age. These cases and controls cohorts were analyzed for the effect allele frequency difference between cases and controls, with the corresponding estimate of the cohort size needed to achieve 80% GWASs statistical discovery power.

**Figure 1:**
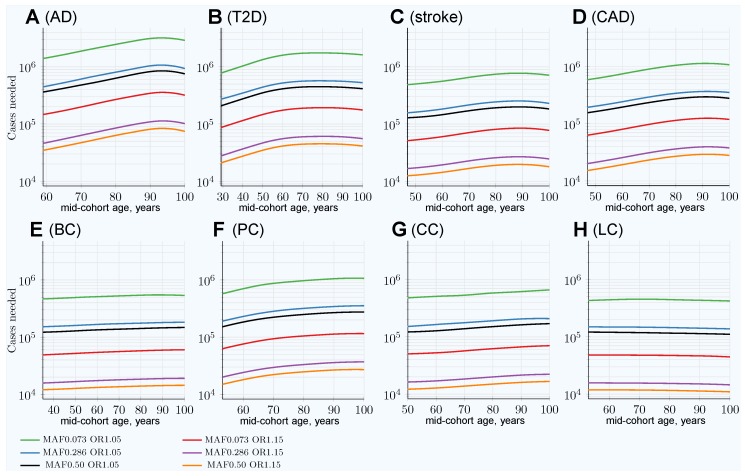
Change in number of cases needed to achieve 80% discovery power in age-matched cases and controls cohort design. (**A**) Alzheimer’s disease (AD), (**B**) type 2 diabetes (T2D), (**C**) cerebral stroke, (**D**) coronary artery disease (CAD), (**E**) breast cancer (BC), (**F**) prostate cancer (PC), (**G**) colorectal cancer (CC), (**H**) lung cancer (LC). Age-matched cohorts require larger numbers of participants to achieve the same genome-wide association studies’ (GWASs’) discovery power compared to the youngest cohort age.

The simulations in [37] determined that the age-related change in the cohort size needed to achieve 80% discovery power for an age-matched case–control cohort study increases with mid-cohort age (with the exception of lung cancer), as presented in Figure 1. This pattern is caused by the diminishing difference in effect SNP frequency between diagnosed cases and unaffected controls as mid-cohort age increases and is also reflected in the decreasing cohort PRS for older cohorts (see Figures S1 and S2). This pattern was consistently observed for all genetic architectures, showing that the change in the PRS depends on the cumulative incidence and the magnitude of heritability (see Table 2). Consequently, the age-matched cohorts composed of the youngest possible participants will allow for the best GWASs’ statistical discovery power compared to older age-matched cohorts.

The number of participants needed to achieve adequate GWASs’ statistical power differs between the lowest- and the highest-effect alleles and also between the lowest and the highest frequency alleles, exhibiting a greater-than-hundredfold variation between alleles composing the genetic architecture, as seen in Figure 1. The required number of cohort participants is quite similar for the same-effect alleles among all eight LODs; for example, the highest-effect allele for each LOD requires $5\times 10^{4}$–$1.4\times 10^{5}$ cases for 80% discovery power at younger ages. The change in allele frequency with age progression between cases and controls shows substantial variation among LODs, with the greatest change occurring in AD and the least significant in lung cancer, as demonstrated in Figure S1.

### 3.2. Advantage of Using Youngest Possible Cases and Oldest Controls in GWASs LOD Cohorts

**Figure 2:**
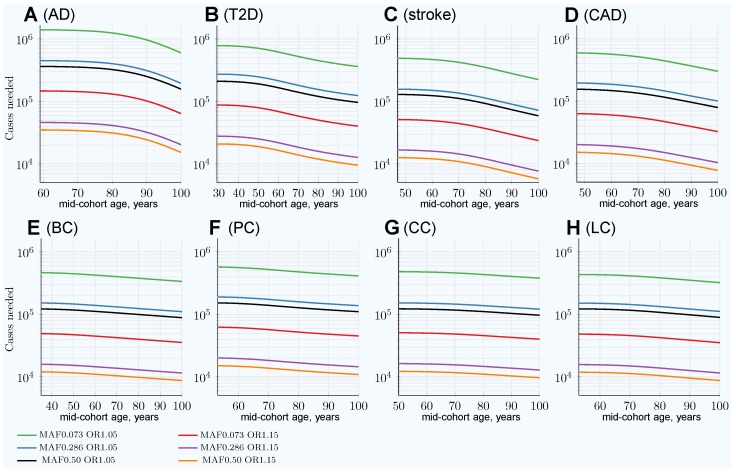
Change in number of cases needed for 80% discovery power in a cohort study when using progressively older controls compared to fixed-age young cases. (**A**) Alzheimer’s disease, (**B**) type 2 diabetes, (**C**) cerebral stroke, (**D**) coronary artery disease, (**E**) breast cancer, (**F**) prostate cancer, (**G**) colorectal cancer, (**H**) lung cancer. Cases’ mid-cohort age is the leftmost age (youngest plot point); control mid-cohort ages are incremental ages. The number of cases needed for 80% discovery power is smaller when using older controls, particularly for those LODs showing the most prominent increase in the number of cases needed for older age in matched-age cohorts, as can be seen in Figure 1.

**Figure 3:**
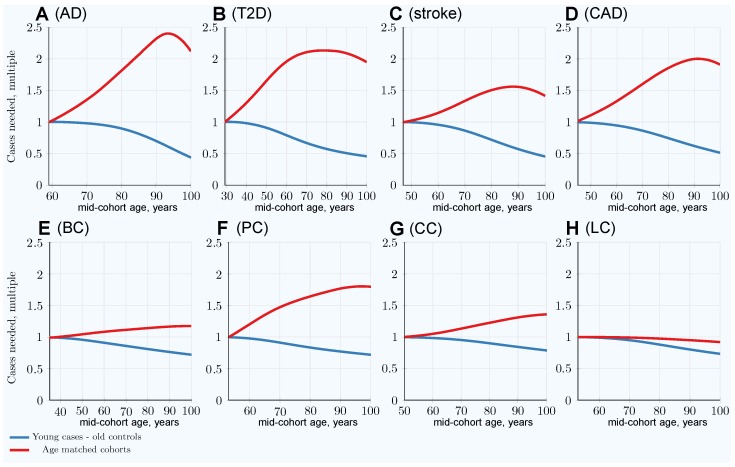
Per LOD comparison: Youngest possible cases and increasingly older controls vs. classical age-matched cohorts. (**A**) Alzheimer’s disease, (**B**) type 2 diabetes, (**C**) cerebral stroke, (**D**) coronary artery disease, (**E**) breast cancer, (**F**) prostate cancer, (**G**) colorectal cancer, (**H**) lung cancer. The multiplier showing the reduction in the number of cases needed in a young cases–older controls scenario is shown in blue (here, cases’ mid-cohort age is the leftmost, the youngest age plot point; control mid-cohort ages are incremental ages), strongly contrasting with the number of cases needed for the same GWASs’ discovery power in a classic age-matched study design, shown in red, which increases with age.

The scenarios simulating the number of cases needed when the cases are the youngest possible participants with increasingly older controls in the cohort are presented in Figure 2. In this scenario, the cohort size to achieve 80% GWASs’ statistical power decreases with the cohort age progression thanks to a change in allele frequency difference between younger cases and older controls cohorts; this is demonstrated in Figure S3. The multiplier representing the decrease in the number of cases that is needed in this scenario is represented by the blue lines in Figure 3, which strongly contrasts with the increasing with age multiplier of the number of cases needed for the same GWASs’ discovery power in the classic age-matched study design demonstrated by the red line.

The age-matched and youngest case/older control scenarios are summarized in Table 2. The youngest cases/older controls cohort scenario multiple was found to be almost identical between all allele frequencies and effect sizes for each particular LOD, as seen in Figure S5. An additional side-by-side view can be seen in Figure S4.

Thus, cohorts composed of the youngest possible cases and the oldest available controls could improve the discovery power of GWASs. Equivalently, such cohorts require even smaller numbers of participants to achieve the same GWASs’ discovery power than the youngest age-matched cohorts, and certainly better than any older age-matched cohorts.

### 3.3. Characterizing and Adjusting for Effect Size in the Younger Cases and Older Controls GWASs

The case–control populations with corresponding individual SNP sets produced by the above simulations where exported in PLINK format [49] for GWAS analysis. Association analysis determined SNP effect sizes and applied iterative age covariate adjustment starting with the youngest possible age-matched cases and controls, and proceeding with the progressively older controls.

The GWASs association analysis with the youngest possible cases and older controls cohorts showed that with the increasing controls cohort age, the SNP effect sizes exceed the known “true” effect sizes. This is the expected consequence of the larger effect allele differential between these cohorts compared with the age-matched cohorts. For SNPs defined in the simulation with the effect size 0.14 (OR = 1.15), the association analysis found effect sizes near 0.20 (OR = 1.21) for CAD and stroke with 100-year-old control cohorts; the bias is notably lower for the four cancers. The differential effect size (bias) of +0.05, corresponding to an OR multiple equal to 1.05, was reached for these LODs at the control group age of 100 years; the bias age progression is displayed in Figures S6 and S7. The typical single-SNP GWASs association analyses are known to show an underestimated SNP effect for higher trait heritablities [63,64]. This is particularly relevant for AD and T2D, with 3575 and 2125 effect SNPs for common low-effect-size genetic architectures. Stringer et al. [63] consider this phenomenon a facet of GWASs’ missing heritability characteristic for single-SNP analysis. Multi-SNP analyses are proposed and are being developed [64,65,66,67]. For the purposes of this study, the customary single-SNP association analysis was sufficient for the relative bias determination for AD and T2D, which was found to closely follow the patterns of CAD and stroke, as well as the cancers.

**Figure 4:**
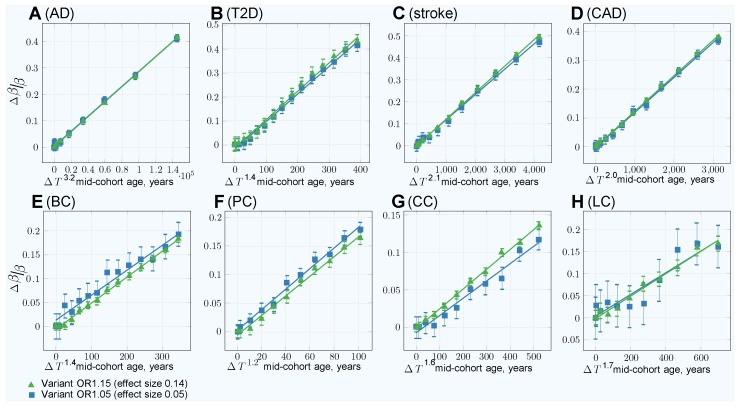
GWASs association simulations: characterizing the age bias adjustment maintaining “true” OR with control cohort age progression (best fit power: $\Delta T^{P}$). (**A**) Alzheimer’s disease, (**B**) type 2 diabetes, (**C**) cerebral stroke, (**D**) coronary artery disease, (**E**) breast cancer, (**F**) prostate cancer, (**G**) colorectal cancer, (**H**) lung cancer. Common, low-effect-size alleles, showing two single nucleotide polymorphisms (SNPs)—with the largest and the smallest effect—for each LOD. The confidence interval bars correspond to two sigma (95%) based on the standard error of linear regression fitting. In this plot, rather than using the square of Δage, the best fit power is iteratively discovered, achieving better residual standard error and *p*-value of the R lm() regression, compared to Figure S8.

The GWASs association analysis and effect-size adjustment with age and corresponding association standard errors are summarized in Table 3; the equations and approach are described in the Materials and Methods, Section 2.3. The progression shape in Figure S7 implies that the bias is proportionate to a power function by age, and the bias magnitude progression appears proportionate to the effect size magnitude. The linear regression fitted the normalized effect bias according to Equations (8) and (9). The best fit power of age regression, with the power exponent specific for each LOD, produced a good match, as can be seen in Figure 4. The quadratic bias adjustment, used by Chatterjee et al. [51], also resulted in a reasonable bias adjustment, as seen in Figure S8. A slight improvement of the best fit power over the quadratic regression means that, for simplicity, the quadratic adjustment will likely be sufficient in practical GWASs bias correction for all LODs analyzed here (compare Figure 4 and Figure S8).

## 4. Discussion

By simulating population age progression under the assumption of relative disease liability remaining proportionate to individual polygenic risk, it was confirmed that individuals with higher risk scores will become ill and be diagnosed proportionately earlier, bringing about a change in the distribution of risk alleles between new cases and the as-yet-unaffected population in every subsequent year of age. With advancing age, the mean polygenic risk of the yet-unaffected aging population diminishes. The fraction of highest-risk individuals diminishes even faster, while at the same time, the LOD incidence increases or remains high with progression of age due to organism aging and cumulative environmental effects. Ultimately, the allele distribution in the as-yet-unaffected population of the same age with a given initial genetic architecture depends solely on cumulative incidence, which represents the fraction of the population that has succumbed to a disease [37]. GWASs’ statistical discovery power is impaired by the change in individual distribution of the PRS at older ages. A larger number of cases and controls is needed at older mid-cohort ages to achieve the same GWASs’ statistical discovery power compared to using younger age-matched cohorts. The effect is most prominent for AD, T2D, stroke, and CAD, which exhibit higher heritability and cumulative incidence compared to the cancers analyzed here. The cancers show a noticeably smaller increase in the number of participants required to achieve the same statistical power, and while other factors could be at play, the probabilistic effects determined by lower incidence and lower heritability of the analyzed most prevalent cancers are sufficient to explain this pattern [37]. Quantitatively, the age-matched cohort studies would require 1.5–2.1 times more participants at age 80 compared to the youngest possible age-matched cohorts in the case of stroke, CAD, AD, and T2D.

Designing cohorts composed of the youngest possible cases and the oldest available controls improves GWASs’ discovery power due to a larger difference in risk allele frequency between cases and controls. This effect is reminiscent of the example given by Sham and Purcell [68] for quantitative traits, in which performing GWASs using only the extreme top and bottom 5% of the individual distribution would achieve the same result with 4.4 times fewer participants compared to a cohort of randomly selected individuals. However, in contrast to the Sham and Purcell [68] example, the observed larger MAF difference here is achieved not because of an enrichment of effect alleles with age—the youngest case–control cohorts show the largest MAF difference and GWASs’ discovery power for the age-matched cohorts—but rather, the MAF difference effect is enhanced by the impoverishment of the increasingly older controls in terms of polygenic risk and corresponding effect allele frequencies. This cohort design leads to a smaller number of participants being needed for GWASs, particularly when applied to the highest cumulative incidence and heritability LODs—so much so that about 50% fewer participants are required to achieve the same GWASs’ statistical power when control cohorts between 90 and 100 years of age are matched to the youngest case cohorts, with the reverse being the case with older age-matched cohorts. Also notably, 20–25% fewer participants are needed in this scenario to achieve the same statistical power in cancer GWASs, including even those focusing on lung cancer.

Use of non-age-matched cases and controls in GWASs cohorts, while improving the discovery power, may result in reporting a higher association effect then the “true” effect, as should be expected with the enhanced difference in the effect of SNP frequency between cases and controls, and would require appropriate adjustment, as demonstrated in the results in Section 3.3. This study’s simulations imply that the adjustment may be simplified by the fact that the bias magnitude was found to be proportionate to the associated SNP effect size. Many GWASs association software packages offer automated covariate bias correction [49,50,69,70,71] and allow for additional scripting.

Not every GWAS will be able to find a sufficient number of youngest cases, as this study used as the basis of comparisons. However, due to a close to exponential rise in the incidence rate with age for most LODs at initial onset ages [37], the case cohorts can be formed at a somewhat older age or with a wider cohort span, with a correspondingly somewhat smaller improvement in GWASs’ discovery power. For all LODs analyzed, a majority of the population would remain disease-free at the ages of 80 and 90 years, with sufficient survivorship to provide a large pool of older controls.

The results of this study are based on idealized simulations assuming that the gene–environment interaction, including the organism deterioration caused by the aging process, follows Cox’s proportional hazards model. The population in these simulations is homogeneous in all respects, while a practical GWAS would always have a varying degree of population diversity and nonhomogeneity that must be accounted for and addressed in a GWAS’ quality control and study design [72]. Additionally, younger patients may be confounded by other health and environment conditions; for example, Boehme et al. [57] showed that concomitant with T2D diagnoses, other LOD incidences may be shifted toward younger ages: by on average 20 years earlier for hypertension, followed by eight years earlier for coronary heart disease onsets, and four years earlier for stroke onsets. Lee et al. [73] linked T2D with earlier or more severe AD manifestation. Studies by Song et al. [40] and Langenberg et al. [42] indicated that the multiplicative (Cox’s) model is applicable in SNP-by-SNP and SNP-by-environment interactions corresponding to the above examples, yet young cases’ quality control may require higher diligence. While these concerns apply to age-matched and youngest possible cases–older controls study designs, the advantage of the latter scenario may be realized to a varying degree in the practical GWASs.

A concept that deserves mention is the effect known as genetic innovation—the idea that certain variants may have a greater effect in older individuals. The concept is most researched in psychiatry, where it is observed primarily during human development; for example, studies of symptoms of anxiety and depression [74,75] demonstrated genetic innovation effects “coming on line” at ages 8–9, 13–14, 16–17, and 19–20, intermixed with phases of the opposite effect called “attenuation”. A review [76] estimated genetic innovation for a range of developmental stages and psychiatric phenotypes. Research is ongoing into mechanisms like methylation with age [77] and modulation with age in transcriptome and exon splicing [78] that may influence DNA configuration and gene expression, and as a result, influence the causal for LODs SNP effect sizes. An interesting approach to finding SNPs associated with hypertension treatment (reminiscent of this study design, except studying a trait quantitatively rather than an LOD diagnosis), where participants were grouped into 10-year cohorts between 20 and 80 years of age, allowed the discovery of mildly protective SNPs [79,80]. As was reviewed in the Introduction, there is a strong theoretical and experimental consensus regarding multiplicative gene–environment genetic interaction explaining the incidence of the diseases analyzed here. Hypothetically, a younger cases–older controls study design with proper quality control may also help to settle the existence of the genetic innovation SNPs when performing routine LOD GWASs, and if promising SNPs were discovered, fine-tune the case–control ages and age direction for more precise determination.

## 5. Conclusions

The simulation results demonstrated that GWASs of the polygenic LODs that display both high cumulative incidence at older ages and high initial familial heritability will benefit from using the youngest possible participants. Moreover, GWASs would benefit from using as controls participants who are as old as possible. This may allow for an additional increase in statistical discovery power thanks to achieving a greater difference in risk allele frequency between cases and controls.

## Figures and Tables

**Table 1:** Heritability of analyzed LODs and an example of required variant numbers for common low-effect variants.

	Highly Prevalent LODs				Cancers			
	AD	T2D	Stroke	CAD	Breast	Prostate	Colorectal	Lung
Heritability	0.795	0.69	0.41	0.55	0.31	0.57	0.40	0.095
SNP number	3575	2125	625	1175	400	1250	600	100

**Table 2:** Comparative summary of the older age-matched cohorts and the youngest cases–older controls cohorts to the youngest age-matched cohorts.

	Highly Prevalent LODs				Cancers			
	AD	T2D	Stroke	CAD	Breast	Prostate	Colorectal	Lung
LOD characteristics:								
Lifetime risk %	10–20	55	25–30	32–49	12	12	< 4.5	<6.9
Heritability %	79–80	69	38–44	50–60	31	57	40	8–18
Maximum yearly incidence %	> 20	2.5	4.4	3.6	<0.5	<0.8	<0.6	<0.6
Cohort size multiple for:								
Age-matched at 80 years	1.82	2.13	1.51	1.86	1.15	1.65 (1.36)	1.25	0.98
Youngest cases & controls at 80 years	0.89	0.57	0.72	0.75	0.81	0.84 (0.82)	0.90	0.88
Relative advantage: 80-year-old controls	2.04	3.74	2.10	2.48	1.42	1.96 (1.66)	1.39	1.11
Cohort size multiple for:								
Age-matched at 100 years	2.12	1.95	1.42	1.91	1.19	1.80 (1.44)	1.36	0.92
Youngest cases & controls at 100 years	0.43	0.46	0.46	0.52	0.72	0.72 (0.70)	0.79	0.74
Relative advantage: 100-year-old controls	4.39	4.24	3.09	3.67	1.65	2.50 (2.06)	1.72	1.24

The minor allele frequency (MAF) values and cases needed for 0.8 (80%) GWASs’ statistical discovery power are for the common, low-effect-size alleles. Simulated cohorts span 10 years. The two comparison blocks for 80-year and 100-year mid-cohort age compare the multiple of cases needed against the youngest possible age-matched cohorts for each late-onset disease (LOD). The values show how many times larger (or smaller) a cohort size the corresponding scenario would require to achieve the same 80% GWASs’ statistical discovery power. Prostate cancer heritability is 57% according to [61]. Shown in braces is 42% heritability [62], which according to [37] is perhaps a more reasonable—although historically older—estimate of prostate cancer heritability and is more in line with the other three cancers.

**Table 3:** Summary of GWASs association simulations and effect size correction parameters for youngest cases–older controls cohorts.

	Highly Prevalent LODs				Cancers			
	AD	T2D	Stroke	CAD	Breast	Prostate	Colorectal	Lung
Youngest cases mid-cohort age	59	29	47	44	35	53	50	53
**GWAS Association SSE for β=0.14 (OR1.15):**								
100Y controls β SSE raw	0.00312	0.00311	0.00312	0.00311	0.00310	0.00310	0.00310	0.00310
100Y controls β SSE adjusted	0.00342	0.00345	0.00359	0.00336	0.00315	0.00314	0.0312	0.00314
**GWAS Association SSE for β=0.05 (OR1.05):**								
100Y controls β SSE raw	0.00283	0.00283	0.00283	0.00283	0.00283	0.00283	0.00283	0.00283
100Y controls β SSE adjusted	0.00310	0.00311	0.00321	0.00304	0.00288	0.00288	0.00285	0.00287
**Age bias adjustment—quadratic ($\Delta age^{2}$):**								
Slope coefficient	$2.3\times 10^{-4}$	$9.1\times 10^{-5}$	$1.8\times 10^{-4}$	$1.2\times 10^{-4}$	$4.4\times 10^{-5}$	$7.7\times 10^{-5}$	$5.5\times 10^{-5}$	$8.1\times 10^{-5}$
Residual standard error	0.029	0.026	0.0058	0.0039	0.0093	0.014	0.0050	0.0092
*p*-value	$5.5\times 10^{-7}$	$1.9\times 10^{-12}$	$2.9\times 10^{-16}$	$2.7\times 10^{-18}$	$1.8\times 10^{-11}$	$4.1\times 10^{-7}$	$2.7\times 10^{-10}$	$5.3\times 10^{-9}$
**Age bias adjustment—best fit power ($\Delta age^{P}$):**								
Power	3.2	1.4	2.1	2.0	1.4	1.2	1.6	1.7
Slope coefficient	$2.8\times 10^{-6}$	$1.2\times 10^{-3}$	$1.2\times 10^{-4}$	$1.2\times 10^{-4}$	$5.4\times 10^{-4}$	$1.7\times 10^{-3}$	$2.6\times 10^{-4}$	$2.6\times 10^{-3}$
Residual standard error	0.0030	0.013	0.0057	0.0039	0.0036	0.0053	0.0025	0.0084
*p*-value	$6.9\times 10^{-15}$	$1.3\times 10^{-16}$	$2.5\times 10^{-16}$	$2.7\times 10^{-18}$	$2.0\times 10^{-16}$	$5.3\times 10^{-11}$	$5.8\times 10^{-13}$	$2.4\times 10^{-9}$

The two *GWASs association* sections summarize the standard error of the logistic regression association for cohort studies with the largest age difference between youngest case group (as specified for each LOD in this table) and a control group with the mid-cohort age of 100 years. The “raw” value corresponds to the analysis without the age bias adjustment and “adjusted”, after the age bias adjustment (SSE - sum of squared errors). The two *Age bias adjustment* sections show parameters of the regression described in the Methods section when using quadratic and best fit power for the age difference between the youngest cases’ mid-cohort age and incrementally increasing the mid-cohort age of older controls for the SNP with “true” OR = 1.15. The best fit power results in a more accurate regression, but the quadratic rule would be sufficiently accurate for the practical GWASs data.

## Supplementary Materials

The following are available online at https://www.mdpi.com/2075-4426/9/3/38/s1, Supplementary Document: A document Supplementary.PDF containing supplementary figures; Supplementary Data: A zip file SupplementaryData.ZIP containing the simulation executable, the source code, R scripts, batch files, and simulation results.

## Abbreviations

The following abbreviations are used in this manuscript:

AD	Alzheimer’s disease
CAD	coronary artery disease
GWAS	genome-wide association study
LOD	late-onset disease
MAF	minor allele frequency; customarily implying the “effect allele frequency”
OR	odds ratio
PRS	polygenic risk score
SNP	single nucleotide polymorphism; in context of this study used synonymously with the term ‘allele’
T2D	type 2 diabetes
