# Supplementary material for: Evaluating the Potential of Younger Cases and Older Controls Cohorts to Improve Discovery Power in Genome-Wide Association Studies of Late-Onset Diseases
Source: J Pers Med. 2019 Jul 22;9(3):38. doi: 10.3390/jpm9030038 (PMC6789773; doi:10.3390/jpm9030038)
Supplement: Supplementary file 1 [file jpm-09-00038-s001.pdf]

Evaluating the Potential of Younger Cases and Older Controls Cohorts to Improve Discovery Power in Genome-wide Association Studies of Late-onset Diseases

Roman Teo Oliynyk[1,2,*]

[1]Centre for Computational Evolution, University of Auckland, Auckland 1010, New Zealand
[2]Department of Computer Science, University of Auckland, Auckland 1010, New Zealand
[*]roli573@aucklanduni.ac.nz

SUPPLEMENTARY FIGURES

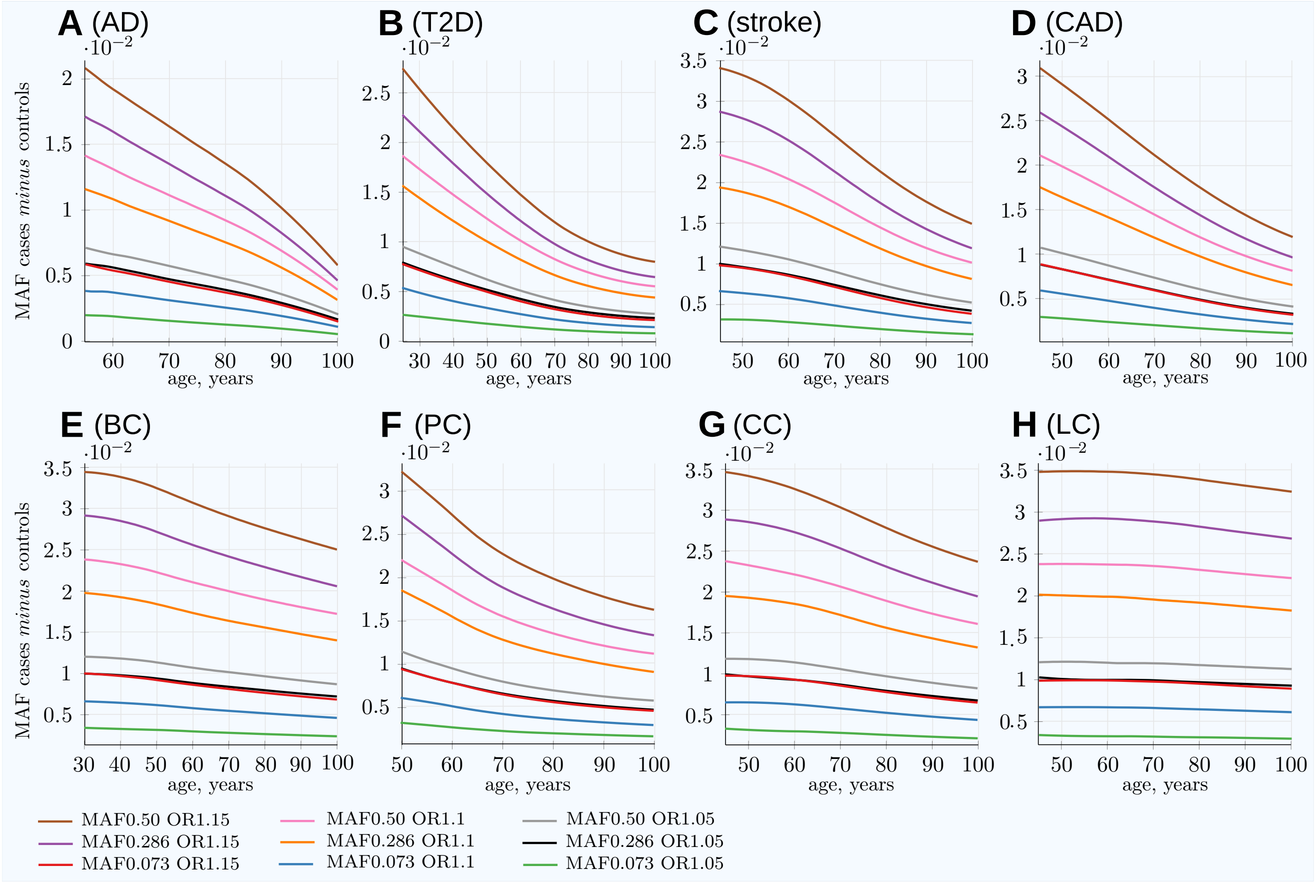

Figure S1. Difference in frequency in allele between newly diagnosed individuals and remaining population of the same age.
(A) Alzheimer's disease, **(B)** type 2 diabetes, **(C)** cerebral stroke, **(D)** coronary artery disease, **(E)** breast cancer, **(F)** prostate cancer, **(G)** colorectal cancer, **(H)** lung cancer.
The **MAF cases *minus* controls** value is used to determine GWASs' statistical power. Rarer and lower-effect-size (OR) alleles are characterized by a lower MAF relative change. *(Displayed here: nine out of 25 SNPs for common low-effect-size genetic architecture)*. From Oliynyk (2019).

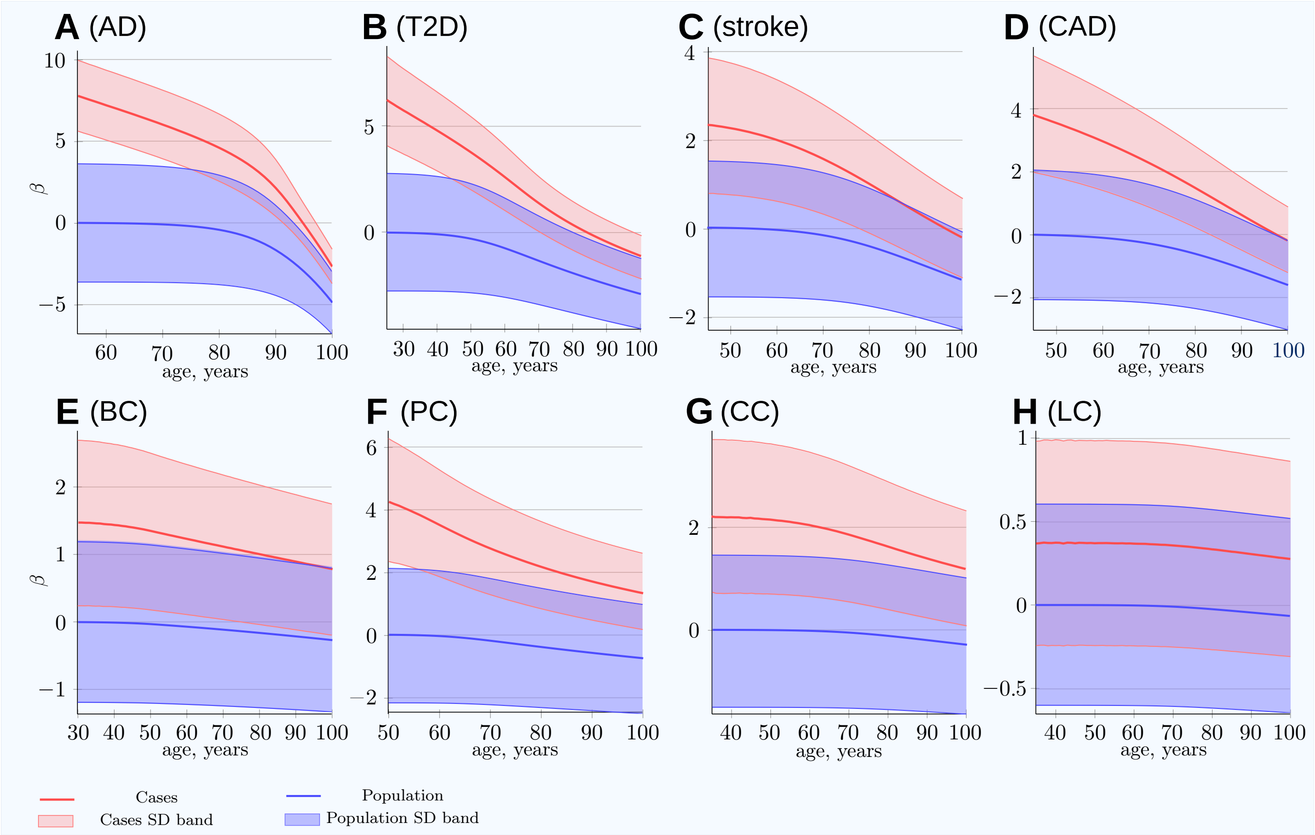

Figure S2. Polygenic risk score difference between newly diagnosed individuals and the remaining unaffected population.

(A) Alzheimer's disease, **(B)** type 2 diabetes, **(C)** cerebral stroke, **(D)** coronary artery disease, **(E)** breast cancer, **(F)** prostate cancer, **(G)** colorectal cancer, **(H)** lung cancer.

SD band is a band of one standard deviation above and below the cases and the unaffected population of the same age. For highly prevalent LODs, at very old age, the mean polygenic risk of new cases crosses below the risk of an average healthy person at early onset age. *(Common low-effect-size alleles, showing largest-effect variant with MAF = 0.5, OR = 1.15).* From Oliynyk (2019).

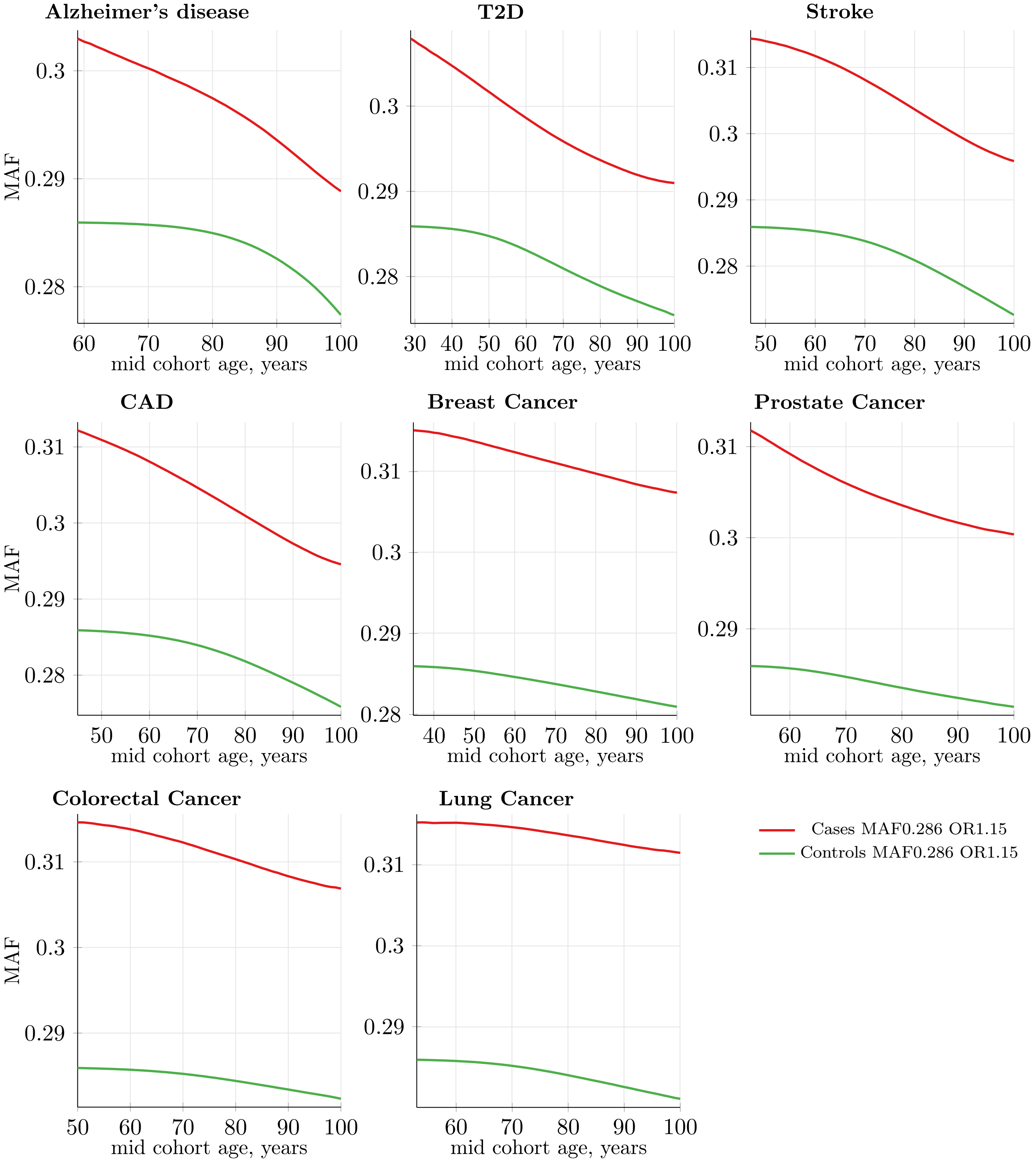

Figure S3. Absolute magnitude change in minor allele frequency (MAF) with age for cases and controls; cohort simulation.

Common, low-effect-size alleles; all plots show MAF = 0.286 and OR = 1.15 allele. Change in the absolute magnitude of each allele frequency value is relatively small with age progression. GWASs' discovery power is a function of the difference in allele frequency between cases and controls.
It is easy to visually estimate the change in the difference in allele frequency between the cases and controls. In the age-matched scenario, the difference is taken between points on the line at the same mid-cohort age. For the youngest cases-older controls scenario, the difference is taken always between the leftmost point on the red line and progressively older controls on the green line. From Supplemental Information in Oliynyk (2019).

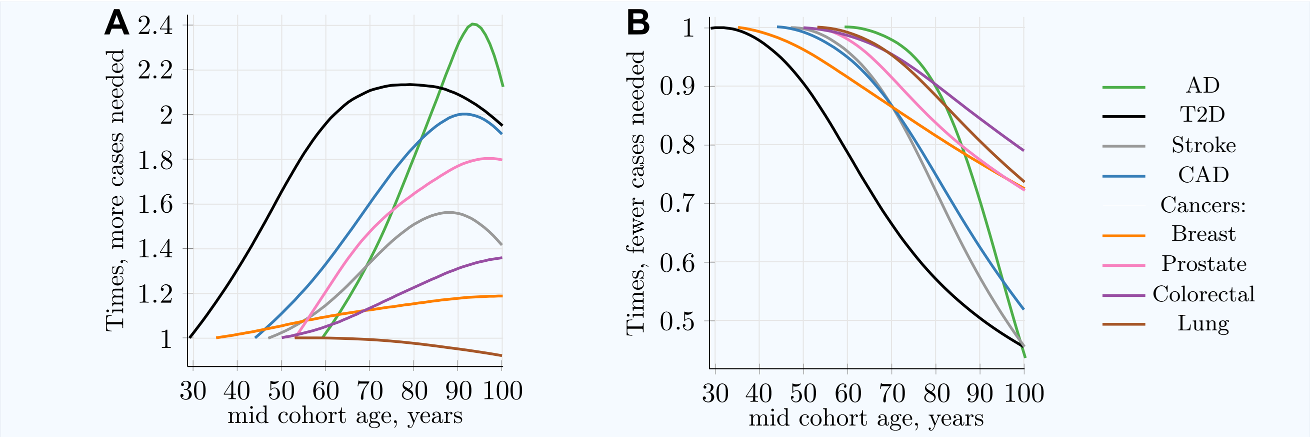

Figure S4. Advantage of using youngest possible cases and increasingly older controls compared to classical age-matched cohorts.
(A) Relative **increase in number of cases needed** for 80% discovery power in a cohort study using progressively older **case and control cohorts of the same age**. **(B)** Relative **decrease in the number of cases needed** for 80% discovery power in a cohort study using **progressively older control cohorts compared to fixed-age young-case cohorts**. The youngest age cohort for each LOD is defined as the mid-cohort age at which the cumulative incidence for a cohort first reaches 0.25% of the population. Therefore, the leftmost point on each LOD line is the reference (youngest) cohort, and as cohorts age, the cohort case number multiple required to achieve 0.8 statistical power is relative to this earliest cohort. While all alleles display a different magnitude of cases needed to achieve the required statistical power, the change in the multiplier with age is almost identical for all alleles within a given genetic architecture scenario. *(Common low-effect-size genetic architecture)* Plot A of this figure from Oliynyk (2019).

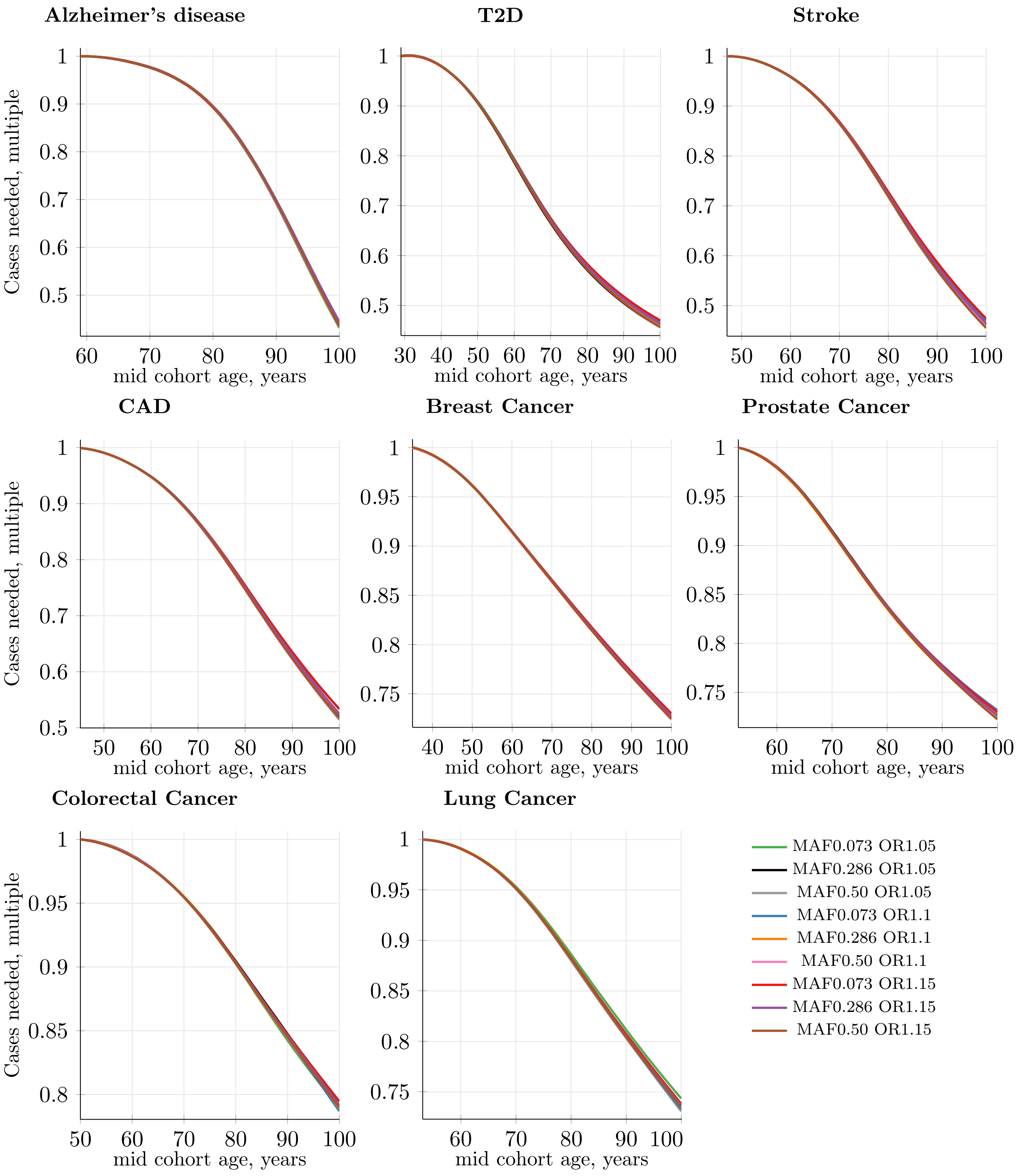

Figure S5. Multiple of the decline in the number of cases needed for 0.8 discovery power in a cohort study using progressively older control cohorts compared to a fixed-age young-cases cohort.
Cases' mid-cohort age is leftmost age (youngest plot point); control mid-cohort ages are incremental ages. The number of cases needed for 0.8 discovery power is smaller when older controls are used, particularly for LODs with the highest heritability and incidence. Common, low-effect-size alleles. A sample of nine out of 25 SNPs; MAF = minor (risk) allele frequency; OR = risk odds ratio.

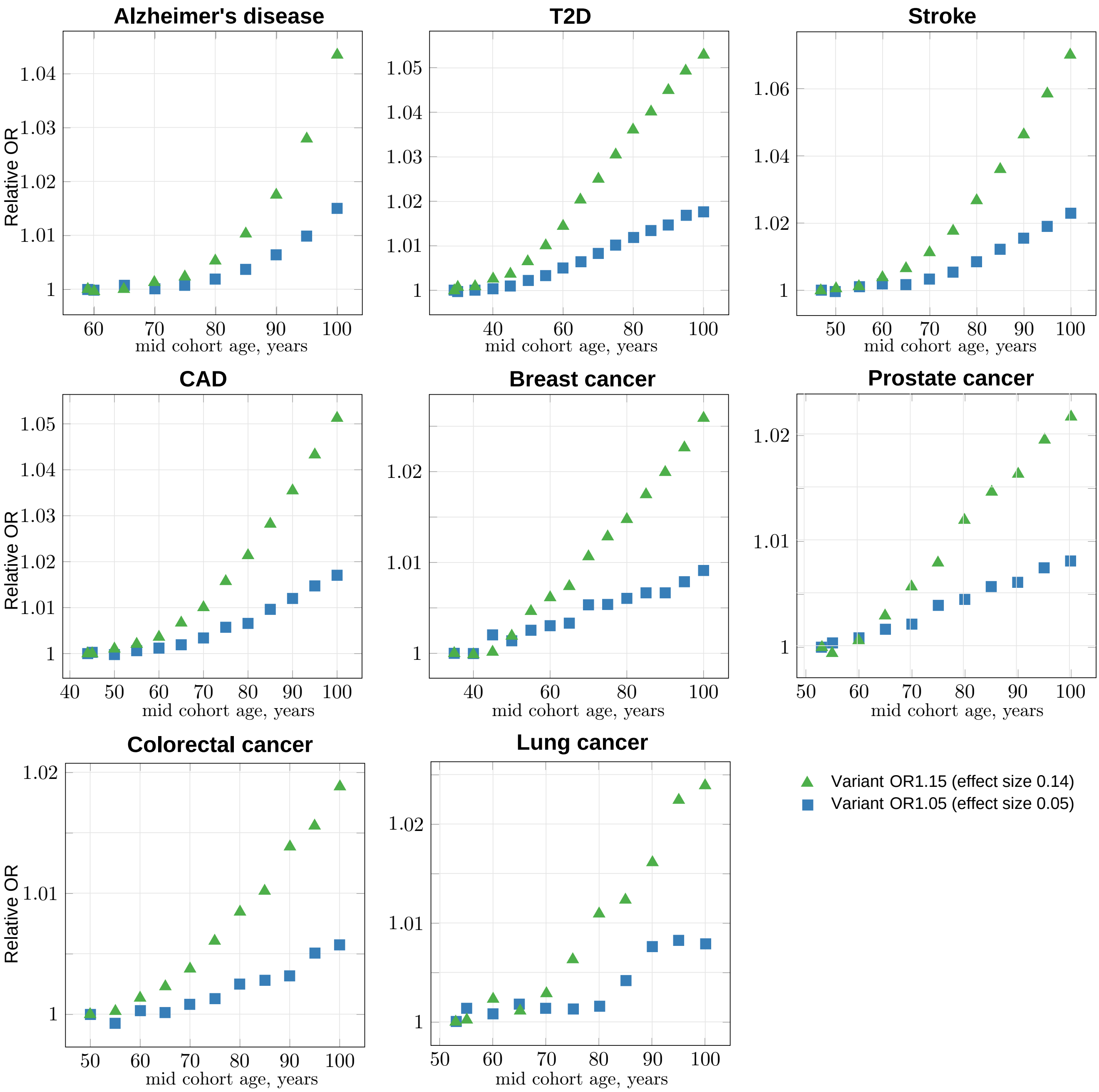

Figure S6. GWAS association simulations: OR bias progression with control cohort age increasing against the constant youngest possible case cohort.
Common, low-effect-size alleles, showing two SNPs—with the largest and the smallest effect—for each LOD. The OR increase (bias) with mid-cohort age progression implies a power of ΔAge from age-matched youngest cohort. The confidence intervals are not displayed on this plot for illustration purposes; they are displayed in Figure S7, showing the same data in effect size units.

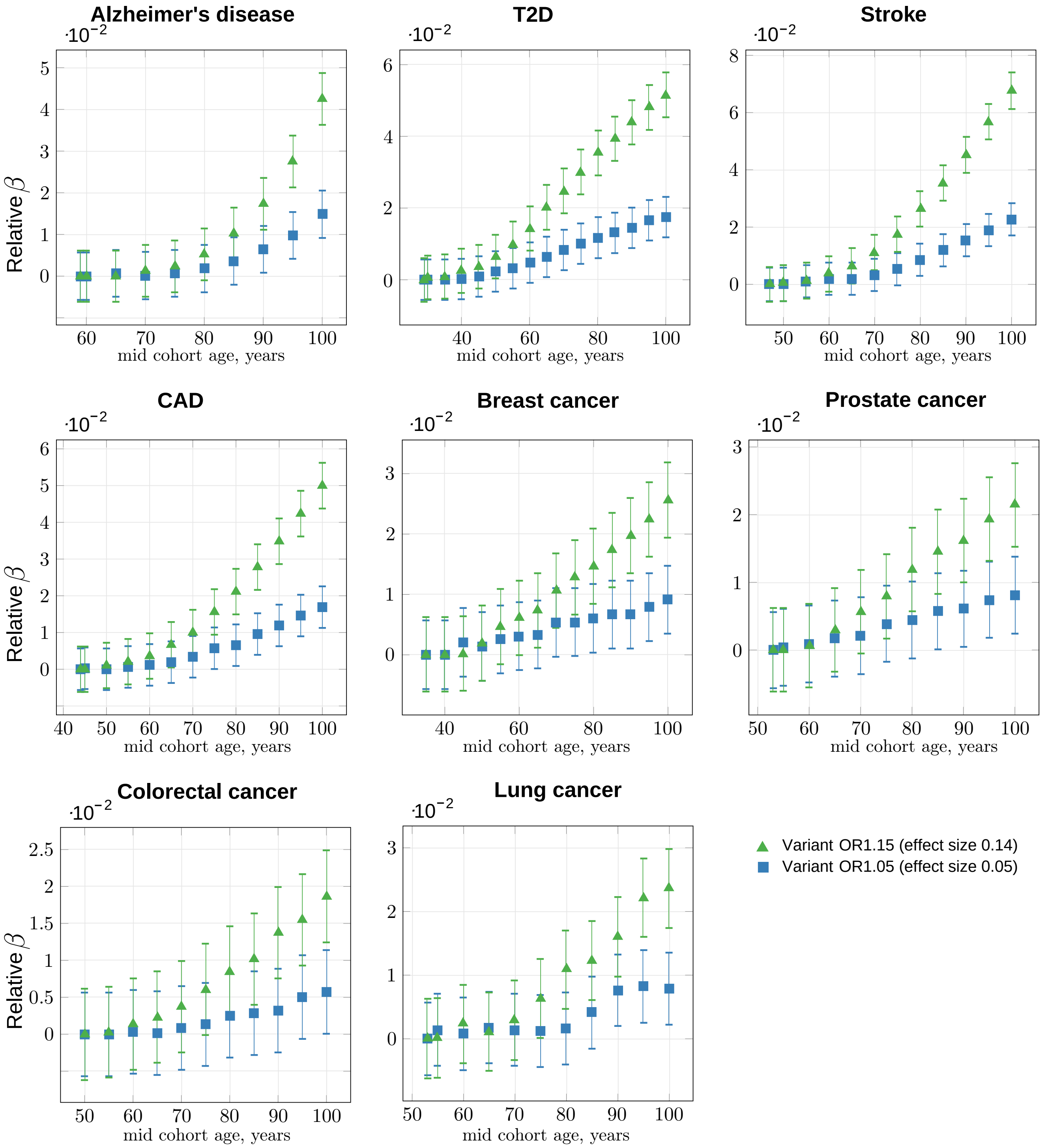

Figure S7. GWASs association simulations: relative effect size bias progression with control cohort age increasing against the constant youngest possible case cohort.
Common, low-effect-size alleles, showing two SNPs—with the largest and the smallest effect—for each LOD. The confidence interval bars correspond to two-sigma (95%) confidence from the GWASs' logistic regression association. The OR increase with mid-cohort age progression implies a power law relative to Δage. This plot implies the LOD SNP age bias and corresponding adjustment value are proportionate to the SNP effect size. For reference, Figure S9 shows absolute effect size progression.

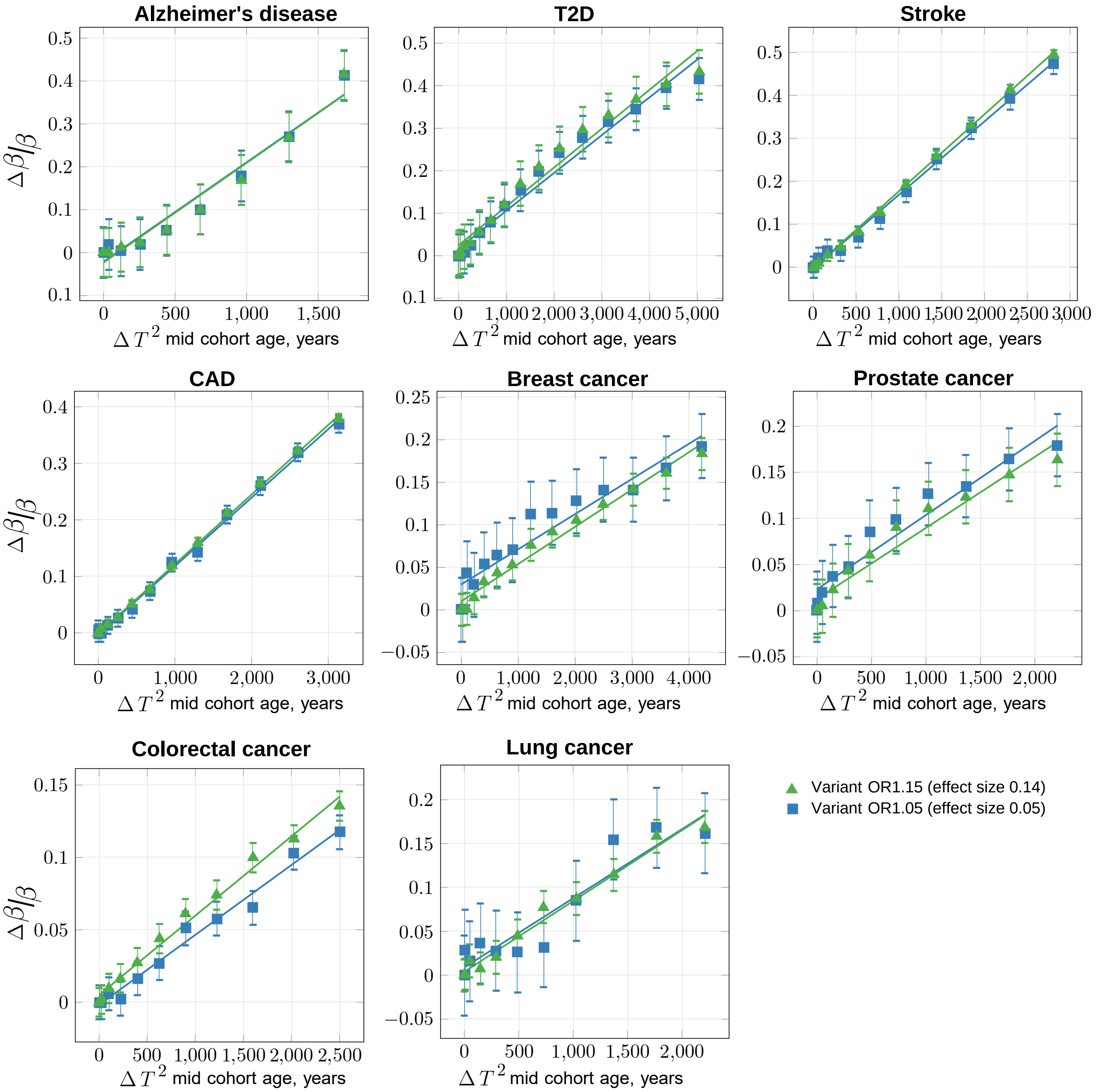

Figure S8. GWASs association simulations: characterizing the age bias adjustment maintaining "true" OR with control cohort age progression (quadratic: ΔT^2).
Common, low-effect-size alleles, showing two SNPs—with the largest and the smallest effect—for each LOD. The confidence interval bars correspond to two-sigma (95%) based on standard error of linear regression fitting.
This plot depicts the adjustment proportionate to square of $\Delta t = t - T_Y$ - relative age from the youngest cohort mid-cohort age for the normalized bias of the effect size β calculated $\Delta\beta/\beta$, as described in the main article.

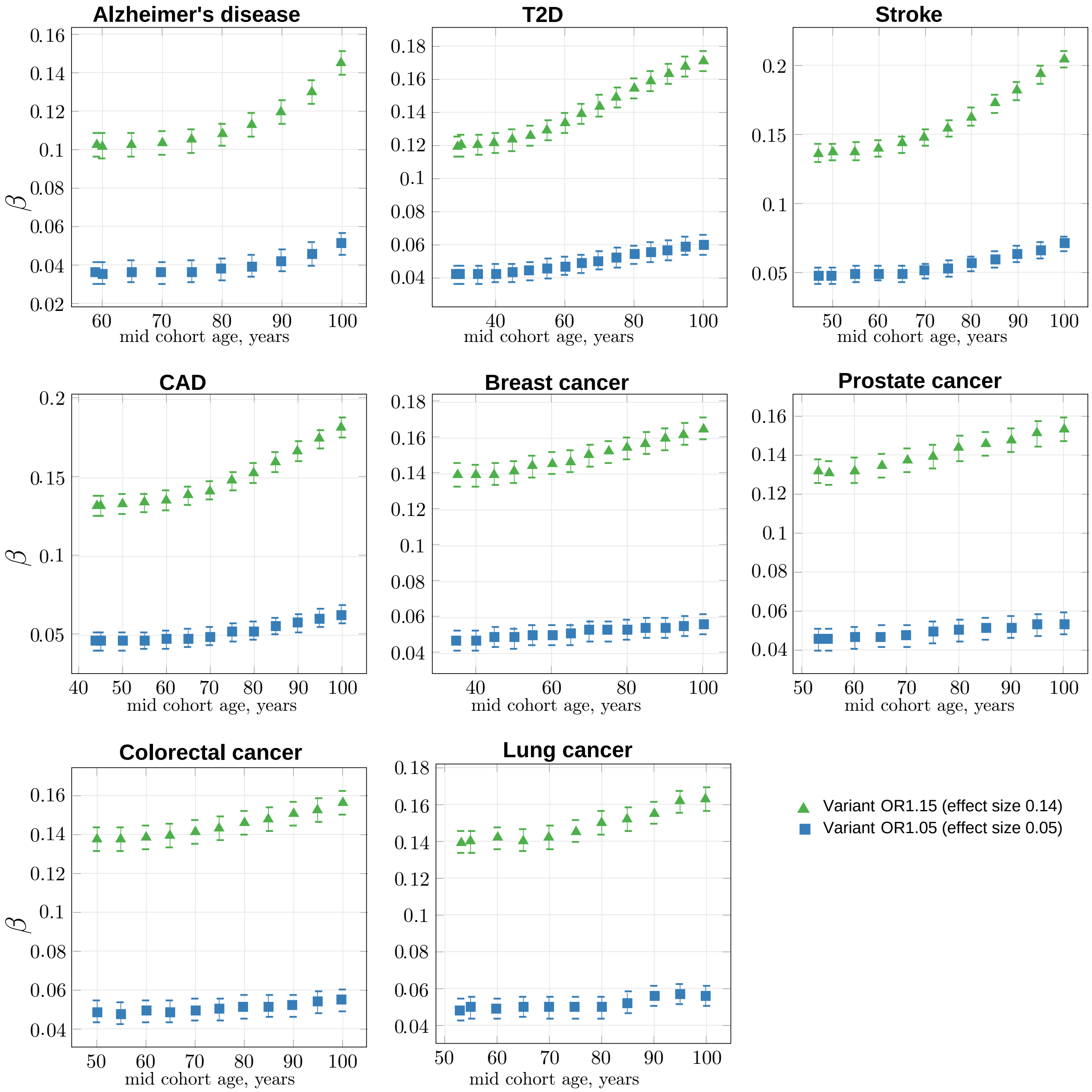

Figure S9. GWASs association simulations: absolute effect size progression with control cohort age increasing against the constant youngest possible case cohort.
Common, low-effect-size alleles, showing two SNPs—with the largest and the smallest effect—for each LOD. The confidence interval bars correspond to two-sigma (95%) confidence from the GWASs' logistic regression association.

References

R. T. Oliynyk. Age-related late-onset disease heritability patterns and implications for genome-wide association studies. *PeerJ*, 7: e7168, Jun 2019. ISSN 2167-8359. doi: 10.7717/peerj.7168.
